# Real-time observation of X-ray-induced intramolecular and interatomic electronic decay in CH_2_I_2_

**DOI:** 10.1038/s41467-019-10060-z

**Published:** 2019-05-16

**Authors:** Hironobu Fukuzawa, Tsukasa Takanashi, Edwin Kukk, Koji Motomura, Shin-ichi Wada, Kiyonobu Nagaya, Yuta Ito, Toshiyuki Nishiyama, Christophe Nicolas, Yoshiaki Kumagai, Denys Iablonskyi, Subhendu Mondal, Tetsuya Tachibana, Daehyun You, Syuhei Yamada, Yuta Sakakibara, Kazuki Asa, Yuhiro Sato, Tsukasa Sakai, Kenji Matsunami, Takayuki Umemoto, Kango Kariyazono, Shinji Kajimoto, Hikaru Sotome, Per Johnsson, Markus S. Schöffler, Gregor Kastirke, Kuno Kooser, Xiao-Jing Liu, Theodor Asavei, Liviu Neagu, Serguei Molodtsov, Kohei Ochiai, Manabu Kanno, Kaoru Yamazaki, Shigeki Owada, Kanade Ogawa, Tetsuo Katayama, Tadashi Togashi, Kensuke Tono, Makina Yabashi, Aryya Ghosh, Kirill Gokhberg, Lorenz S. Cederbaum, Alexander I. Kuleff, Hiroshi Fukumura, Naoki Kishimoto, Artem Rudenko, Catalin Miron, Hirohiko Kono, Kiyoshi Ueda

**Affiliations:** 1 Institute of Multidisciplinary Research for Advanced Materials, Tohoku University, Sendai, 980-8577 Japan; 2 RIKEN SPring-8 Center, Sayo, Hyogo 679-5148 Japan; 3 Department of Physics and Astronomy, University of Turku, Turku, FI-20014 Finland; 4 Department of Physical Science, Hiroshima University, Higashi-Hiroshima, 739-8526 Japan; 5 Department of Physics, Kyoto University, Kyoto, 606-8502 Japan; 6 Synchrotron SOLEIL, L’Orme des Merisiers, Saint-Aubin, BP 48, FR-91192 Gif-sur-Yvette, Cedex France; 7 Department of Chemistry, Graduate School of Science, Tohoku University, Sendai, 980-8578 Japan; 8 Department of Physics, Lund University, Lund, SE-22100 Sweden; 9 Institut für Kernphysik, J.W. Goethe Universität, Max-von-Laue-Str. 1, D-60438 Frankfurt, Germany; 10 School of Physics and Nuclear Energy Engineering, Beihang University, Beijing, 100191 P.R. China; 11 Extreme Light Infrastructure - Nuclear Physics (ELI-NP), “Horia Hulubei” National Institute for Physics and Nuclear Engineering, RO-077125 30 Reactorului Street, Măgurele, Jud Ilfov Romania; 12 National Institute for Laser, Plasma and Radiation Physics, 409 Atomistilor, PO Box MG-36, 077125 Magurele, Jud Ilfov Romania; 13 European XFEL GmbH, Holzkoppel 4, Schenefeld, D-22869 Germany; 14 ITMO University, Kronverksky pr. 49, St. Petersburg, 197101 Russia; 15 Institute of Experimental Physics, Technische Universität Bergakademie Freiberg, 09599 Freiberg, Germany; 16 Institute for Materials Research, Tohoku University, Sendai, 980-8577 Japan; 17 Japan Synchrotron Radiation Research Institute (JASRI), Sayo, Hyogo, 679-5198 Japan; 18 Theoretical Chemistry, PCI, Universität Heidelberg, 69120 Heidelberg, Germany; 19 ELI-ALPS, Budapesti út 5, H-6728 Szeged, Hungary; 20 J. R. Macdonald Laboratory, Department of Physics, Kansas State University, Manhattan, KS 66506 USA; 21 LIDYL, CEA, CNRS, Université Paris-Saclay, CEA Saclay, 91191 Gif-sur-Yvette, France

**Keywords:** Chemical physics, Free-electron lasers, Atomic and molecular interactions with photons

## Abstract

The increasing availability of X-ray free-electron lasers (XFELs) has catalyzed the development of single-object structural determination and of structural dynamics tracking in real-time. Disentangling the molecular-level reactions triggered by the interaction with an XFEL pulse is a fundamental step towards developing such applications. Here we report real-time observations of XFEL-induced electronic decay via short-lived transient electronic states in the diiodomethane molecule, using a femtosecond near-infrared probe laser. We determine the lifetimes of the transient states populated during the XFEL-induced Auger cascades and find that multiply charged iodine ions are issued from short-lived (∼20 fs) transient states, whereas the singly charged ones originate from significantly longer-lived states (∼100 fs). We identify the mechanisms behind these different time scales: contrary to the short-lived transient states which relax by molecular Auger decay, the long-lived ones decay by an interatomic Coulombic decay between two iodine atoms, during the molecular fragmentation.

## Abstract (web summary)

Understanding strong X-ray induced phenomena is important for applications of X-ray free-electron laser imaging. Here, the authors show time-resolved measurements of X-ray free-electron laser induced electronic decay of CH_2_I_2_ molecule probed with NIR pulses and identify mechanisms behind different transient states lifetimes.

## Introduction

Understanding the details of the interaction between intense X-ray free-electron laser (XFEL) pulses^1,2^ and matter is of paramount importance for its numerous applications, including single-particle structural determination by coherent X-ray imaging^3–5^ and structural dynamics tracking in molecules by time-resolved X-ray spectroscopy and diffraction^6–12^. Another powerful method made available by the new XFELs is serial femtosecond crystallography^13–15^. It allows structural determination of proteins, especially membrane proteins, which are difficult to crystalize. However, an in-depth knowledge of the radiation damage caused by the XFEL irradiation^16–19^ is necessary for the implementation of the above-mentioned XFEL-based methods. In imaging applications, the primary purpose of the XFEL pulses is to probe the structure of the object, ideally as noninvasively as possible. The impact of the extremely concentrated energy of the XFEL pulses on the target materials is, however, although severe, still not fully characterized. In particular, the changes induced in the electronic structure of the sample in the course of the imaging process are poorly known. A prominent example is the application of XFEL imaging to complex molecular photocatalysts containing heavy metal atoms. In such photocatalysts, the deep inner-shell ionization of heavy atoms releases many electrons via cascading electronic relaxation processes and, owing to the Coulomb repulsion between highly charged atomic sites, eventually results in the fast destruction of chemical bonds and consequently in rapidly developing radiation damage at the molecular level. Detailed knowledge of the underlying mechanisms is obtained through real-time observations of the structural changes and of the charge states, resulting from the interrogation of the sample by the XFEL pulses.

The first studies of the decay processes triggered by the interaction of XFEL pulses with atoms and molecules have been performed at both the Linac Coherent Light Source up to 2 keV photon energy^20–22^ and at 7 keV photon energy^23^, and the SPring-8 Angstrom Compact free-electron LAser (SACLA) in the 5–15 keV photon energy range^24–28^, and focused in particular on small organic molecules and biomolecules containing iodine atoms as strong X-ray absorption centers. Specifically, when iodine-containing molecules are irradiated by high-energy 5.5 keV XFEL pulses, iodine 2p subshell photoionization occurs first with the highest probability^26^. Then, additional positive charges are produced locally at the iodine site by Auger decay cascades. In the final stages of these cascades, involving delocalized molecular orbitals, the positive charges redistribute over the entire molecule and a highly charged molecular cation is formed. This molecular ion undergoes Coulomb explosion into mostly atomic fragment ions.

Our former studies applied an empirical charge and nuclear dynamics model to the experimental XFEL data and concluded that the charge generation and redistribution are ultrafast, taking place within the XFEL pulse duration (∼10 fs), in competition with the Coulomb explosion^26,27^. These conclusions were drawn indirectly, by comparing the asymptotic predictions of the model with the final charge, energy, and momentum distributions of the observed Coulomb explosion products. However, to directly observe the time evolution of these quantities triggered by the initial photoionization event, time-resolved pump-probe measurements are indispensable.

In the following, aiming to better understand the molecular-level radiation damage in matter containing heavy atoms, we study diiodomethane (CH_2_I_2_) by time-resolved ion momentum spectroscopy. The CH_2_I_2_ molecule results the substitution of two hydrogens with two iodine atoms in methane, and may be seen as the simplest model system of that type. We experimentally determine the lifetimes of the transient states populated during the XFEL-induced Auger cascades and find that multiply charged iodine ions are issued from short-lived (∼20 fs) transient states, whereas the singly charged ones originate from significantly longer-lived states (∼100 fs). Our investigation allows us to identify the driving mechanisms behind: contrary to the short-lived transient states which relax by molecular Auger decay, the long-lived ones decay, during the molecular fragmentation process, by an interatomic Coulombic decay (ICD) process involving two iodine atoms.

## Results

### Investigated process

**Fig. 1:**
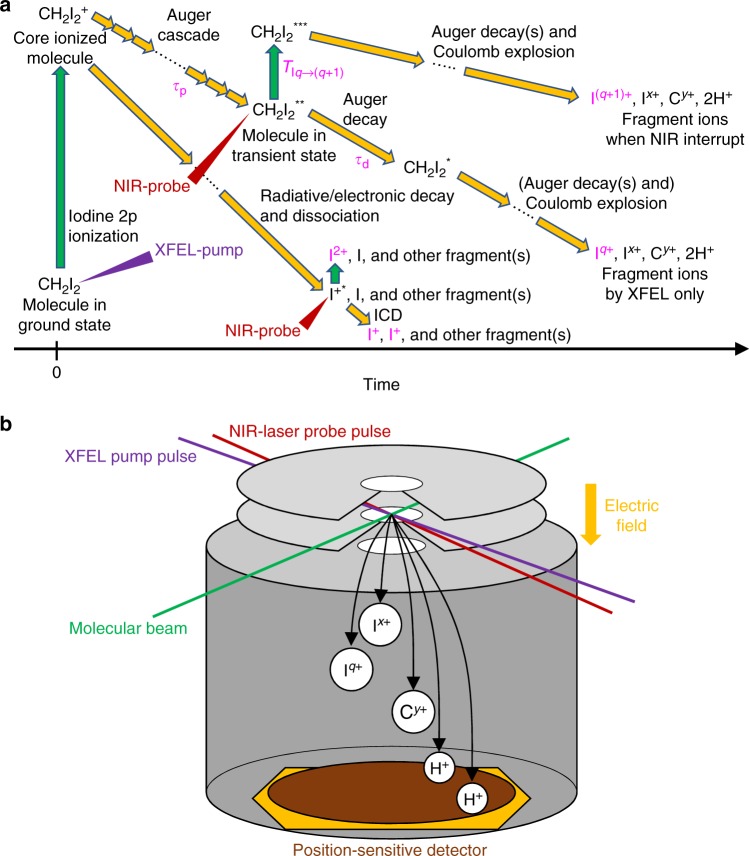
Schematics of the experiment. **a** Diagram of the XFEL-induced processes and the NIR-induced effects. The process starts from iodine 2p ionization by the XFEL-pump pulse irradiation, creating the short-lived 2p^−1^ state. This moment is defined as the time origin. The molecular cation then starts to dissipate energy by an Auger cascade, which is a step-wise process populating intermediate transient electronic states of increasingly higher charge. The cascade, if uninterrupted, continually lowers the electronic internal energy of the molecule. However, a NIR-probe pulse can interfere with the normal course of the cascade decay and excite the molecule (CH_2_I_2_^**^) to a higher energy level (CH_2_I_2_^***^). This process, in order to occur with high probability, may require the molecule to be in a suitable electronically excited state when the NIR-probe pulse arrives. We denote the population time and the lifetime (depopulation time) of such an excited state as *τ*_p_ and *τ*_d_, respectively. After such a NIR-induced step-up in energy, the Auger cascade can proceed via higher energy levels, eventually resulting in a higher total charge than would have been reached without the additional NIR-probe energy. Also, the NIR-pulse can directly ionize the molecule to the next-higher charge state, from where the Auger cascade continues, again reaching a higher final charge. We denote as *T*_I*q*→(*q*+1)_ the increase of the I^(*q*+1)+^ ion yield owing to the NIR-laser absorption by the transient states (CH_2_I_2_^**^) that would yield I^*q*+^ without NIR-laser interruption. In addition, if a pair of an excited I^+*^ and a neutral iodine atom is produced, ICD may be possible and two I^+^ ions are resulting. When excited I^+*^ is ionized by the NIR-probe, an I^2+^ ion is produced and the neutral iodine remains. **b** Experimental configuration. The XFEL-pump pulse and NIR-laser probe pulse cross at a focal point of both lasers. The molecular beam crosses both lasers at the focal point. The ions released from the molecule by the XFEL/NIR-laser irradiations are accelerated by an electric field and detected by a position-sensitive detector

Figure 1a outlines the processes investigated in the present pump-probe experiment. We used 5.5 keV XFEL pulses to induce decay processes in CH_2_I_2_, and near-infrared (NIR) optical laser pulses as a probe. The pulse durations of the XFEL and NIR-laser are ∼10 fs and 32 fs, respectively. The quantity that can be directly extracted for each charge state *q* of iodine from the recorded ion yields is the delay-dependent variation of the population of next-higher charge state *q* + 1, induced by the NIR probe. The *T*_I*q*→(*q*+1)_ curves defined in Fig. 1a directly reflect the temporal evolution of the transient electronic states described by the population time *τ*_p_ and the lifetime *τ*_d_, allowing us to extract these quantities directly from our time-resolved measurement.

### Charge state distributions

**Fig. 2:**
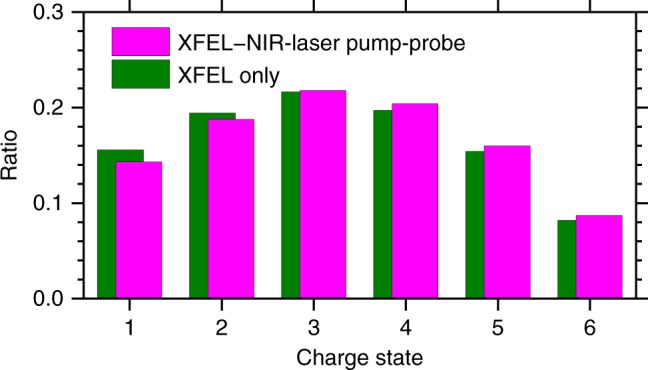
Charge state distributions of iodine ions. Green bars indicate the yield of various iodine ions charge states produced by XFEL radiation only, normalized to the sum of the I^+^–I^6+^ yields. Magenta bars show the yields obtained when the NIR probe was added to the XFEL pulse, within a delay time window between −45 fs and +125 fs. The figure thus does not yet differentiate between the various delay times, but presents the overall effect of adding the NIR pulse

In the present experiment, we measured momentum vectors of the released fragments by a time-of-flight (TOF) spectrometer equipped with a position-sensitive detector as drawn in Fig. 1b. Details of the experiment are described in Methods. Figure 2 shows the charge state distributions of iodine ions with and without the NIR-laser irradiation. In either case, the distribution peaks at a charge state of +3. The relative abundance of I^3+^ does not change when the NIR laser is added, whereas for the higher charge states it increases when the NIR laser is added, and whereas the opposite occurs for lower charge states, which are depleted by the NIR pulse. This result is consistent with the overall picture that a population transfer from lower to higher charge states occurs for the iodine ions owing to the additional excitation by the NIR-probe, as described in Fig. 1a. In the experiment, we also observed carbon ions. However, as the XFEL-induced electronic decay dynamics in Fig. 1a are observable mainly in the iodine ion yields, we focused on the behavior of the iodine ions. For a sake of completeness we present the charge state distributions for carbon ions in the Supplementary Fig. 1 and comment them in the Supplementary Note 1.

### Time-evolution of ion yields

**Fig. 3:**
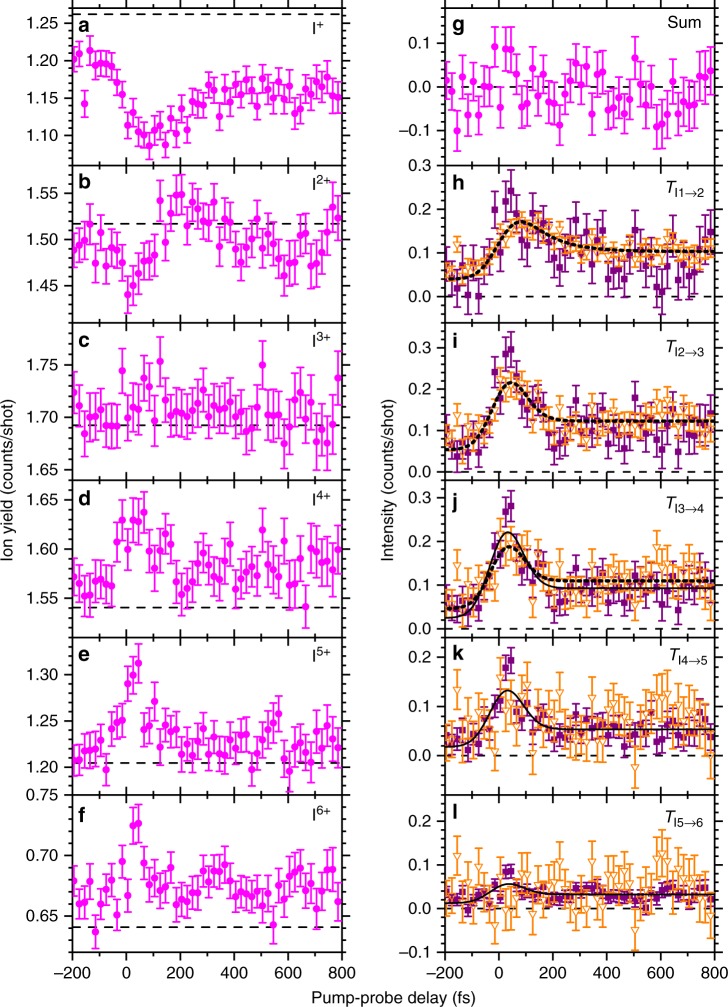
Time evolution of the iodine ion yields. **a**–**f** Iodine ion yields plotted as a function of the pump-probe delay. Magenta full circles indicate the pump-probe dependence of the I^*q*+^ ion yield, *Y*_I*q*_(*t*), and the horizontal dashed lines indicate the baselines of the I^*q*+^ ion yields, *B*_I*q*_. We obtained *B*_I*q*_ from the sum of the ion TOF spectra measured using the XFEL-only and NIR-only ionization (the latter only produced the I^+^ ions). **g** Sum of (*Y*_I*q*_(*t*) − *B*_I*q*_) for *q* = 1–6. **h**–**l** *T*_I*q*→(*q*+1)_(*t*) obtained using equations (3) (purple full squares) and (4) (orange empty triangles). Solid lines and dotted lines indicate the fitted curves for purple full squares and orange empty triangles, respectively, with *τ*_*p*_ = 10 fs. As both data sets in **h**–**l** represent the same quantity *T*_I*q*→(*q*+1)_(*t*), we used the sets with better statistics for the fitting, namely, the purple full squares in **h**–**j** and the orange empty triangles in **j**–**l**. Error bars are defined as standard deviation

Figure 3a–f illustrates the pump-probe delay (*t*) dependence of the I^*q*+^ yields, *Y*_I*q*_(*t*), where *q* designates the charge state. Baselines, that is the I^*q*+^ yields generated by single-pulses (either XFEL or NIR), *B*_I*q*_, are also shown for reference. Deviations from the baseline, *Y*_I*q*_(*t*) − *B*_I*q*_, are thus attributed to the pump-probe combined effect and can be represented as a dynamic balance of the inflow to and the outflow from a given charge state I^*q*+^:

1 $$Y_{{\mathrm{I}}q}(t) - B_{{\mathrm{I}}q} = T_{{\mathrm{I}}(q - 1) \to q}(t) - T_{{\mathrm{I}}q \to (q + 1)}(t).$$

We can obtain the target quantities *T*_I*q*→(*q*+1)_(*t*) straightforwardly from the measured yields *Y*_I*q*_(*t*) and *B*_I*q*_ as shown below. From equation (1), we obtain

2 $$\mathop {\sum}\limits_{q = 1}^6 \left( {Y_{{\mathrm{I}}q}(t) - B_{{\mathrm{I}}q}} \right) = T_{{\mathrm{I}}0 \to 1}\left( t \right) - T_{{\mathrm{I}}6 \to 7}(t).$$

Figure 3g depicts the left side of equation (2). There, we do not see any significant delay dependence and the values are close to zero, indicating that both *T*_I0→1_(*t*) and *T*_I6→7_(*t*) are negligibly small. One can then obtain all other curves *T*_I*q*→(*q*+1)_(*t*): if *T*_I6→7_(*t*) = 0, then *Y*_I6_(*t*) − *B*_I6_ = *T*_I5→6_(*t*) and

3 $$T_{{\mathrm{I}}q \to (q + 1)}(t) = \mathop {\sum}\limits_{n = q + 1}^6 \left( {Y_{{\mathrm{I}}n}(t) - B_{{\mathrm{I}}n}} \right),$$

for *q* ≤ 5. If *T*_I0→1_(*t*) = 0, then *Y*_I1_ − *B*_I1_(*t*) = −*T*_I1→2_(*t*) and

4 $$T_{{\mathrm{I}}q \to (q + 1)}(t) = \mathop {\sum}\limits_{n = 1}^q \left( {B_{{\mathrm{I}}n} - Y_{{\mathrm{I}}n}(t)} \right),$$

for *q* ≥ 1. In Fig. 3h–l, we plot *T*_I*q*→(*q*+1)_(*t*) for *q* = 1 − 5 obtained using both equations (3) and (4). The good agreement between these two plots within the error bars confirms that we successfully extracted the target quantities *T*_I*q*→(*q*+1)_(*t*).

## Discussion

There are two components in the temporal behavior of *T*_I*q*→(*q*+1)_(*t*) in Fig. 3h–l: one is a peak structure that appears near 0 fs and the other one is a step-increase at positive delay. The peak structure is related to the interaction of the NIR probe with transient electronic states formed in the intermediate stages of the Auger cascade, that is, the target process described in Fig. 1a. The step-increase maintaining the NIR-induced effect at the asymptotic limit is, on the other hand, a result of the NIR probe interacting with the Coulomb explosion ionic fragments. We carried out curve-fitting analysis taking into account these two contributions. The details are provided in Methods. In the discussion below, we focus on the peak structures to which the target processes contribute. For completeness, the step structures are also explained in the Supplementary Note 1.

**Fig. 4:**
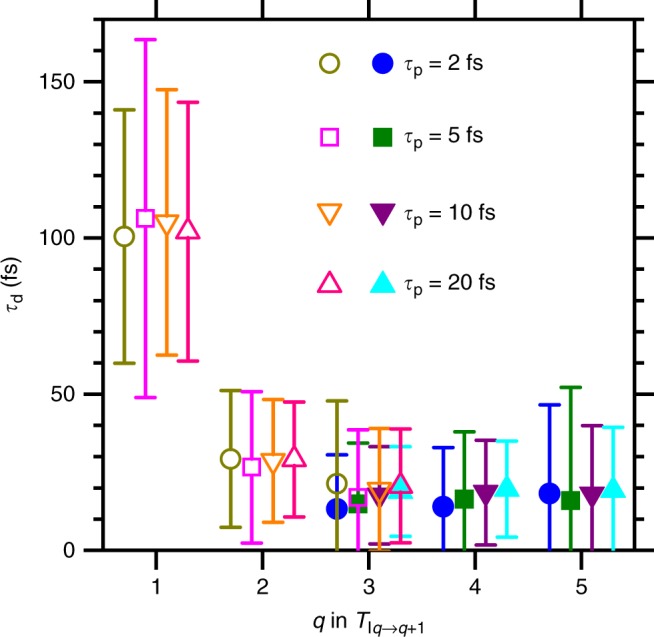
Decay time constants. *τ*_d_ obtained from the fitting for *T*_I*q*→(*q*+1)_(*t*) based on equation (3) (full symbols) and equation (4) (empty symbols). The fittings were performed using *τ*_p_ = 2 fs, 5 fs, 10 fs, and 20 fs. Error bars are defined as standard deviation

We used a fitting model with the short populating times *τ*_p_ as pre-determined parameters, allowing also to investigate the sensitivity and variability of the other fitted parameters. The population of the transient state can be treated as being completed within the XFEL pulse duration^28^. Therefore, *τ*_p_ was considered to be ≤10 fs. Figure 3 illustrates the fitted curves when *τ*_p_ is set to be 10 fs. Figure 4 shows the decay times *τ*_d_ obtained from the fitting using *τ*_p_ = 2, 5, 10, and 20 fs. The *τ*_d_ extracted from the fitting for each *q* should not be considered as lifetimes of specific electronic states but rather as representative values for all transient states contributing to each *T*_I*q*→(*q*+1)_(*t*) channel. One can see that *τ*_d_ is not sensitive to the choice of *τ*_p_. Notably, *τ*_d_ becomes much smaller for *T*_I2→3_(*t*) compared with *T*_I1→2_(*t*), and then remains unchanged for higher charge states. This sensible shortening of the decay time constant of the molecular transient states indicates that a significant change occurs in the electronic decay pathway of these states as the charge state increases.

Whereas the pump-probe delay dependence of the carbon ions yields may include information about the charge transfer from the iodine to the carbon site as shifted step structures^29^, we were unable to extract such information because of the overlap between peak and step structures. We present the time evolution of the carbon ions yields in the Supplementary Fig. 2 and explain it in the Supplementary Note 1, for completeness.

Let us now concentrate on the remarkable feature that for *T*_I1→2_(*t*), *τ*_d_ is significantly longer than any other (*q* ≥ 2) decay time constant. For the latter, *τ*_d_ are consistent with the typical electronic state lifetimes in the intermediate stages of the Auger cascades^23,24^. For the former, however, *τ*_d_ is too long to be associated with typical inner-shell vacancy lifetimes. Instead, the NIR-pulse interaction with these long lifetime transient states producing low-charge iodine ions may be affected by the molecular dissociation process. In the 100-fs timescale, both hydrogen and carbon atoms may be significantly away (≥10 Å) from the two iodine atoms, whereas the two iodine atoms may still be close to each other (≤6 Å)^28^. We therefore interpret this slow decay as an ICD process^30^ where an electronically excited iodine ion (e.g., I^+^(5p^3^5d)) decays, whereas the neutral neighbor iodine is ionized. See the Supplementary Fig. 3 for detail. ICD following Auger decay was first observed in argon dimers^31^, investigated for various systems since then, and recognized as an ubiquitous phenomenon in clusters irradiated with high-energy photons^32^. The arrival of the NIR-probe pulse quenches this ICD channel, the excited iodine ion being further ionized, whereas the neutral iodine remains untouched. Such an ICD channel is energetically open even at equilibrium I–I distance in the neutral CH_2_I_2_, and its rate slows down as the separation of the two sites increases^33^. The measured lifetime of ∼100 fs is comparable with the previous direct measurements of the ICD decay time^34,35^. Therefore, the very different decay time scales of the transient states probed here can be attributed to either fast molecular Auger cascades or to slow ICD between fragments.

To further confirm our conclusion, we performed ab initio calculations of the ICD decay widths of three excited cationic states of the system I^+*^–I as a function of the interatomic distance (see Supplementary Fig. 4). The results show that the lifetimes vary from 2–20 fs, at the I–I distance of 3.5 Å (close to the equilibrium I–I distance in the neutral CH_2_I_2_), to a few hundred fs when the distance becomes 6 Å. In view of the large number of such states populated in the cascade and along the dissociation dynamics, an averaged ICD lifetime ∼100 fs is, therefore, very reasonable. Our calculations indicate that ICD processes in the I^+*^–I system producing two I^+^ are indeed possible and that the experimentally observed time constant of ∼100 fs matches well these decay mechanisms.

When we consider the contribution of ICD, deviations from the baseline for I^+^ need to include the outflow from I^+^ to neutral iodine, *T*_I1→0_(*t*): *Y*_I1_(*t*) − *B*_I1_ = *T*_I0→1_(*t*) − *T*_I1→2_(*t*) − *T*_I1→0_(*t*). As the sum of (*Y*_I*q*_(*t*) − *B*_I*q*_) for *q* = 1–6 is zero, indicating that *T*_I0→1_(*t*) − *T*_I1→0_(*t*) is negligible, ICD does not change equation (4) and thus does not affect the above discussions.

Although we could extract time scales of XFEL-induced electronic decay dynamics using only the iodine ion yields, it was important to also include carbon ions to fully understand how the NIR-probe influences ion yields. As a result of the NIR-probe, not only increments in the ionic charge states, but also energy shifts of the fragment ions owing to the increase of the Coulomb repulsion were observed. To investigate variations in energy, kinetic energy filtered ion yields have been plotted as a function of the pump-probe delay (Supplementary Note 2 and Supplementary Fig. 5). The complete details of the role of the NIR-probe described in Fig. 1a are given in the Supplementary Note 3 with the help of Supplementary Fig. 6. This detailed understanding of the role of the NIR-probe pulse enforces and validates the present analysis.

In conclusion, we measured charge and kinetic energy selected ion yields obtained from the CH_2_I_2_ molecule in an XFEL–NIR-laser pump-probe experiment. We extracted the lifetimes of the transient states produced by the interaction with the XFEL pulse and we found that the lifetimes become notably shorter when the iodine ion charge state increases above *q* = 1. We further revealed the underlying mechanism, and namely that the transient states, which produce I^+^ decay slowly, reflecting an I–I ICD process, whereas the shorter lifetimes measured for the higher charge states are the fingerprint of faster Auger decay cascades. Note that we already established an approach to probe molecular structures by ion momentum correlation measurements using the same apparatus as used here^26–28^. The general approach and the present success in directly probing in real-time XFEL-induced transient states surviving only tens of femtoseconds indicate that we established a tool to observe ultrafast XFEL-induced reactions when we combine time-resolved ion momentum measurements and ion momentum correlation measurements, thus inducing a research scope in XFEL science.

## Methods

### Experiment

The experiments were carried out at the experimental hutch 4 (EH4) of beamline 3 (BL3) of SACLA^36–38^. The XFEL pulse and optical laser pulse were used as pump and probe pulses, respectively.

The XFEL beam was focused by a Kirkpatrick–Baez mirror system^39^ to a focal size of ∼1 μm (full width at half maximum; FWHM) in diameter. The repetition rate of the XFEL pulses was 30 Hz. The photon energy was set at 5.5 keV and the photon bandwidth was ~20 eV (FWHM). The pulse duration of the XFEL was not measured but was estimated to be ~10 fs (FWHM)^40^. XFEL pulse energies were measured using a beam position monitor^41^ located upstream of the beamline. That beam position monitor was calibrated by a calorimeter, so that output signals from the monitor could be transformed into the absolute value of the pulse energy^42^. The measured value during this experiment was 5.7 × 10^2^ μJ per pulse on average. The shot-to-shot pulse energy fluctuation was about ± 10% (21% FWHM). Note that the pulse energy is not measured at the reaction point but upstream, and that losses occur due to the beam transport and diagnostics. The peak fluence of the XFEL pulse at the reaction point was 30 μJ μm^−2^ on average. The absolute value of the peak fluence was calibrated just before the experiment by a well-established calibration procedure using argon^24,43^.

The optical laser system synchronized with the XFEL pulses is permanently installed at the beamline^37^. We used NIR pulses with 800-nm wavelength (1.55-eV photon energy). The pulse duration of the NIR laser was measured to be 32 fs (FWHM) and the peak fluence was 11 nJ μm^−2^ (3.3 × 10^13^ W cm^−2^ peak intensity).

In the XFEL–NIR-laser pump-probe measurement, the arrival timing monitor^44^, a kind of cross-correlator between the XFEL and NIR-laser pulses, was used. Originally, the difference between the XFEL pulse and the NIR-laser pulse arrival times had ∼700 fs (FWHM) temporal jitter. After correcting the jitter by using the arrival timing monitor, we binned the data points every 20 fs. In this way, we could achieve a total time resolution of ∼100 fs. Only when such a jitter correction is applied, real-time observation of the ultrafast intramolecular electronic decay processes by using XFEL and optical laser pulses can become reality.

CH_2_I_2_ vapor seeded in helium gas was introduced to the focal point of the XFEL pulses as a pulsed supersonic molecular beam. CH_2_I_2_ with 99.7% purity was purchased from Nacalai Tesque, Inc. and used without further purification. The molecular beam was crossed with the focused XFEL and NIR-laser beams at the focusing point (Fig. 1b). The yields and the three components of the momentum vectors of released ions were measured as a function of pump-probe delay by the TOF type ion spectrometer equipped with a delay-line type position-sensitive detector^45^. We used velocity-map-imaging electric field conditions^46^, in order to obtain high momentum resolution. Signals from the detector were recorded by a digitizer and analyzed by a software discriminator^47^.

### Curve fitting

From Fig. 1a, the peak structure is expected to be formed by the transition to a certain transient electronic state and its decay process^48^. The rising time of the peak corresponds to the population time *τ*_p_, which is necessary to populate a certain transient electronic state during the Auger cascade after iodine 2p photoionization. The tail of the peak structures reflects the decay time *τ*_d_ of this transient electronic state. We performed fitting by considering a Gaussian instrumental response function *g*(*t*) with *σ* being the width of the Gaussian. The temporal trace of the population of the transient state was represented by a populating and depopulating double exponential function *f*(*t*) with two time constants *τ*_p_ and *τ*_d_, respectively:

5 $$f(t) = \left\{ {\begin{array}{*{20}{l}} 0 \hfill & {\left( {t \,< \,t_0} \right),} \hfill \\ {A_{\mathrm{p}}\left[ {1 - {\mathrm{exp}}\left( { - \frac{{t - t_0}}{{\tau _{\mathrm{p}}}}} \right)} \right]{\mathrm{exp}}\left( { - \frac{{t - t_0}}{{\tau _{\mathrm{d}}}}} \right)} \hfill & {\left( {t \ge t_0} \right),} \hfill \end{array}} \right.$$

where *t*_0_ is the origin of the pump-probe delay and *A*_p_ is a constant. From our previous study^28^, the population of the transient state can be treated as being completed within the XFEL pulse duration. Therefore, *τ*_p_ was considered to be ≤ 10 fs. The double exponential function *f*(*t*) was convoluted with a Gaussian function *g*(*t*). Finally, the fitting function *F*(*t*) can be described as the sum of the above convoluted function and one error function *E*(*t*) to take into account the difference of baseline between the positive and the negative delay regions. The width of the error function is fixed to the one of *g*(*t*). The explicit forms of *F*(*t*), *g*(*t*), and *E*(*t*) are given by:

6 $$F(t) = (f \ast g)(t) + E(t) + C,$$

7 $$g(t) = \frac{1}{{\sqrt {2\pi } \sigma }}{\mathrm{exp}}\left( { - \frac{{t^2}}{{2\sigma ^2}}} \right),$$

8 $$E(t) = \frac{{A_{\mathrm{s}}}}{2}\left( {1 + {\mathrm{erf}}\left( {\frac{{t - t_0}}{{\sqrt 2 \sigma }}} \right)} \right),$$

where *C* and *A*_s_ are constants.

By treating *τ*_p_ as pre-determined parameter, we could determine the other parameters from global fitting to *T*_I1→2_(*t*), *T*_I2→3_(*t*), *T*_I3→4_(*t*), *T*_I4→5_(*t*), and *T*_I5→6_(*t*) in Fig. 3. The fitting procedures with *τ*_p_ = 10 fs allowed us to determine the origin of the time delay with an accuracy of 14 fs as a standard deviation and the width of the instrumental function of 141 ± 14 fs (FWHM, that is *σ* = 60±6 fs). For comparison, the zero-delay positions were obtained to be −4 ± 18 fs, +4 ± 18 fs and +12 ± 13 fs and the width of the instrumental function to be 139 ± 15 fs, 135 ± 15 fs and 141 ± 31 fs when we used *τ*_p_ = 20 fs, 5 fs and 2 fs, respectively. *τ*_d_ is not sensitive to the choice of *τ*_p_, as shown in Fig. 4.

### Calculation of ICD decay widths

The Fano–Stieltjes procedure we used for computing the ICD widths of ionization satellite (two-hole one-particle) states of I_2_ is described in detail in refs. ^49,50^. In this approach, the electronic configuration space of the problem is divided into the subspace *P* that comprises of continuum-like configurations, which correspond to a free electron and an energetically accessible final state of the dication, and the subspace *Q*, which comprises of bound-like configurations corresponding to an electron moving in the field of the energetically forbidden final state of the dication. The decay width is given by

9 $${\mathrm{\Gamma }}(E) = 2\pi \mathop {\sum}\limits_\beta |\langle \chi _{\beta \varepsilon }|\hat H_{PQ}|{\mathrm{\Phi }}\rangle |^2$$

where $\hat H$ is the electronic Hamiltonian, |Φ〉 is the bound-like part of the resonance, and |*χ*_*βε*_〉 is the continuum part of the resonance, which describes an outgoing free electron of energy *ε* in the channel *β*. Both the bound and continuum parts of the resonance are obtained by numerical diagonalization of the projected Hamiltonians $\hat H_{QQ}$ and $\hat H_{PP}$. As square-integrable Gausssian type orbitals are used in representing the many-electron wavefunctions, we used the Stieltjes imaging procedure^51,52^ to ensure the proper normalization of |*χ*_*βε*_〉 to energy.

To partition the configuration space, we first used the non-relativistic second-order algebraic diagrammatic construction [ADC(2)] method for the two-particle propagator^53^ to compute the spectrum of doubly ionized I$_2^{2 + }$ states. In this way, we determined the number of the two-site I^+^(5p^−1^) − I^+^(5p^−1^) states, which represent the open ICD channels, as well as of the one-site I − I^2+^(5p^−2^), I − I^2+^(5p^−1^ 5 s^−1^), and I − I^2+^(5 s^−2^) states, which are not accessible in ICD. This information is used by the Fano–Stieltjes routine to construct the continuum and bound parts of the resonance state. The construction was done using extended ADC(2) method for the Green’s function^54^. The width was then computed for a number of adiabatic resonance I − I^+*^(5p^−2^nl) states. We used the restricted Hartree-Fock reference state in our ADC calculations. The molecular orbitals and two-electron integrals were computed using Molcas^55^ quantum chemistry program suite. The calculations were performed with cc-pwCVTZ-PP/ECP basis sets^56,57^, augmented by one s-type, one p-type, and one d-type Kaufmann–Baumeister–Jungen functions^58^.

The large number of and the mutual interactions among the resonance states of I − I^+*^(5p^−2^nl) character, as well as the limited accuracy of the ADC(2)x method in computing the energies of ionization satellites, preclude their precise assignment at the interatomic distances of interest. For example, in the ^2^Π symmetry we obtained 22 one-site satellite states whose ionization energies lie below the lowest one-site double ionization I − I^2+^ threshold and which can decay by ICD. We selected three states and computed their ICD widths. The width of the state with the lowest energy is one order of magnitude larger than the one of the two higher excited satellites, which was previously observed for the ICD of ionization satellites in rare gas dimers^59–61^. The computed ICD lifetime of the lower energy state in the 3.5 Å to 7 Å range is 2 fs to 82.5 fs, whereas for the higher energy satellites it is 20 fs to 1.7 ps. Importantly, the widths noticeably decrease with the interatomic distance as they ought to do for an interatomic decay process.

## Supplementary information

Supplementary Information

Peer Review File

## Data Availability

All relevant data are available from the corresponding author on request.
