# Supplementary material for: Real-time observation of X-ray-induced intramolecular and interatomic electronic decay in CH2I2
Source: Nat Commun. 2019 May 16;10:2186. doi: 10.1038/s41467-019-10060-z (PMC6522627; doi:10.1038/s41467-019-10060-z)
Supplement: Supplementary file 1 — Supplementary Information [file 41467_2019_10060_MOESM1_ESM.pdf]

Supplementary Information

Real-time observation of X-ray-induced intramolecular and interatomic electronic decay in CH_2I_2

Fukuzawa et al.

Supplementary Note 1: Charge state distribution and time-evolution of carbon ions

In the experiment, carbon ions, C^{+}, C^{2+}, C^{3+}, and C^{4+}, were also observed. The charge state distributions of carbon ions are shown in Supplementary Figure 1. Green bars indicate the yield of various charge states produced by the XFEL radiation only, normalized to the sum of the C^{+}–C^{4+} yields. Pink bars show the yields obtained when the NIR probe was added to the XFEL pulse, within a delay time window between –45 fs and +125 fs. The relative population of C^{+} decrease whereas those of C^{3+} and C^{4+} increase in the presence of both XFEL and NIR laser.

The pump-probe delay dependences of the C^{+}, C^{2+}, C^{3+}, and C^{4+} ion yields $Y_{\mathrm{C1}}(t)$, $Y_{\mathrm{C2}}(t)$, $Y_{\mathrm{C3}}(t)$, and $Y_{\mathrm{C4}}(t)$, respectively, are shown in Supplementary Figure 2a–d. The baselines of the C^{q+} ion yield ($B_{\mathrm{C}q}$), shown as dashed horizontal lines, were obtained from the measurement using the XFEL-only ionization. We define $T_{\mathrm{C}q\rightarrow(q+1)}(t)$ as the inflow from C^{q+} to $C^{(q+1)+}$ like in the case of iodine ions. Taking into account that C^{5+} ions are not observed, we can obtain $T_{\mathrm{C}q\rightarrow(q+1)}(t)$ similar to equation (3) in main text:

$$T_{\mathrm{C}q\rightarrow(q+1)}(t) = \sum_{n=q+1}^{4} (Y_{\mathrm{C}n}(t) - B_{\mathrm{C}n}), \tag{1}$$

for q = 0–3. $T_{\mathrm{C}q\rightarrow(q+1)}(t)$ obtained using Supplementary Equation (1) are shown in Supplementary Figure 2e–h. In the case of carbon, the inflow from neutral carbon is not negligible. There are no clear peak structures except in $T_{\mathrm{C0}\rightarrow 1}(t)$. We performed the curve fitting for $T_{\mathrm{C}q\rightarrow(q+1)}(t)$ using the same function used for the iodine ions, $T_{\mathrm{I}q\rightarrow(q+1)}(t)$, with τ_{p} = 10 fs, and t_0 and σ were fixed to the values obtained from the fitting for $T_{\mathrm{I}q\rightarrow(q+1)}(t)$. Results of the fitting indicate the possibility of the existence of the weak peak in $T_{\mathrm{C1}\rightarrow 2}(t)$ and $T_{\mathrm{C2}\rightarrow 3}(t)$. The estimated uncertainty of the values

of τ_{d} is, however, large. Thus, we will use only the intensity of the peak structure after gaussian convolution in the later discussion. The peak structure being small indicates that the contributions of the molecular transient states to increase in the carbon charge are small. In other words, when the charge increases by excitation of the molecular transient states by the NIR pulse absorption, the iodine only increases its charge.

Supplementary Note 2: Kinetic energy filtered ion yields

For each ion, apart from the charge state, also its kinetic energy (KE) could be determined from the three measured momentum components. The KE distributions can then be obtained for ions in each charge state, as was done in Supplementary Reference 1. Then, the temporal behavior of the ions in various parts of the KE distribution can be investigated separately. Supplementary Figure 5 shows the ion yields as a function of the pump–probe delay obtained by separating the KE distribution for each charge state into two KE filtered components: the low and the high ion KE populations. The baseline for each component was subtracted from the data. For each charge state, the median energy in the KE distribution measured by XFEL irradiation only was chosen as the separation point into the two components. The separation into the low and high KE components exposes temporal variations in all ion yields, even for I^{3+}, C^{+}, and C^{2+} ions whose ion yields showed no variation prior to KE filtering (Figure 3c and Supplementary Figure 2a,b).

Supplementary Note 3: Processes caused by the NIR-laser probe pulse

Let us analyze what happens due to the NIR laser irradiation. First, we focus on the late delay region (>300 fs). In this delay region, the contributions from the transient states are completed and isolated fragments are ionized by the NIR laser. When the low KE ions of the charge state q are ionized by the NIR laser, these contribute to increments of the low KE ions of the charge state of $(q + 1)$ because the KEs of ions in charge state q are, owing to the weaker Coulomb repulsion, distributed lower than those in charge state $(q + 1)$. On the other hand, when the yields of the high KE ions in charge state of q increase, those came from the high KE ion in charge state $(q - 1)$. In order to investigate those contributions, we estimated the variations from the baselines in the KE filtered ion yields (Supplementary Figure 5) at the late delay region by the values $(A_\mathrm{s} + C)$ obtained by the same fitting method as used in Figure 3 and Supplementary Figure 2, and summarized the values in frames in Supplementary Figure 6a. The values are indicated in units of 0.01 counts/shot. For the high KE component of I^{2+} ions in which the fitting does not work as shown in Supplementary Figure 5, an average value between 500 fs and 1000 fs is used to estimate the variation from the baseline. The values in parentheses labeled "in" and "out" with positive and negative sign are expected amounts of inflow and outflow, respectively, using the values $(A_\mathrm{s} + C)$ obtained from the fitting when we considered as above. The values in the arrows at the left hand side of the frames are estimated flows obtained by the fitting for Figure 3h–l and Supplementary Figure 2e–h. The values in the Supplementary Figure 6a are consistent with each other within 0.01 counts/shot. We found that decrements of the high KE components in the charge state q contribute to increments of the low KE components in the charge state $(q + 1)$ at low charge states,

whereas decrements of the high KE components contribute increments of the high KE components at higher ($q \geq 3$ before the NIR absorption) charge states for both iodine and carbon ions. This is due to the ratio of increment of the Coulomb repulsion energy, which became smaller when the charge states increased. Namely, for example, in the case of q = +1 $\rightarrow$ +2, the ratio is 2 whereas in the case of q = +5 $\rightarrow$ +6, the ratio is 1.2.

Next, let us consider structures at early pump–probe delay region (<200 fs) in Supplementary Figure 5. Peak or dip structures can be seen in this region. Those are due to the transient states. So far, we discussed about the case when the charge states of detected ions are changed due to the NIR absorption by the molecule in the transient states or the fragments. In order to understand the observed peak and dip structures, we must consider also the case where the charge states of detected ions did not change but the charge states of other ions from the same parent molecule changed. Based on this, let us discuss the significance of variations of the KE filterd ion yields with the help of Supplementary Figure 6b.

When we compare fragment ions of the same charge state, the low KE ions are released from parent ions in low charge states and the high KE ions are released from parent ions in high charge states [1]. The transient state molecules which lead to the low charge parent ions have low ionization potential and those electronic states are dense. Such transient state molecules are considered to easily absorb the NIR laser pulse. Thus, the low KE ions in charge state q decrease by the NIR laser absorption ("out" in blue characters in Supplementary Figure 6b). At the same time, the amount of the charge state $(q + 1)$ increase. Since the Coulomb repulsion energy was smaller

before NIR absorption, the KE of the NIR ionized ions will be lower compared to that of the ions of charge state $(q+1)$ produced by XFEL only. Thus, the amount of low KE ions of charge state $(q+1)$ increase ("in" in blue characters). When the charge states of detected ions do not change but other ions from the same parent molecule change, the KE distributions of the detected ions are shifted to higher KE due to the increase in the Coulomb repulsion energy. As a result, the amounts of low KE ions decrease ("out" in magenta characters) and those of high KE ions increase ("in" in magenta characters). As considered above, whether there is a peak or dip in the low KE plots (blue empty squares in Supplementary Figure 5) determined the summation of "in" and "out". The peak should appear in the high KE plots (magenta full triangles in Supplementary Figure 5). However, there are no clear peaks visible for the high KE ions of I^+ and I^{2+}. This can be explained by the fact that the parent ions which produce I^+ and I^{2+} ions also absorb the NIR pulse because those charge states are low.

Based on the above discussion, we estimated the maximum/minimum values of the peak/dip components using the parameters obtained from the fitting and summarized in frames in the Supplementary Figure 6b. The unit of the values corresponds to 0.01 counts/shot. The values in the arrows at the left hand side of the frames are the estimated flow obtained from the fitting for Figure 3h–l and Supplementary Figure 2e–h. Values in parentheses labeled "in" and "out" could be determined using the values obtained from the fitting as the amounts of inflow and outflow, respectively, to be consistent with the experimental results within 0.02 counts/shot.

As stated above, we were able to fully explain the NIR-probe contribution to the observed

results.

Suppprementary Reference

1. Takanashi, T. *et al.* Ultrafast Coulomb explosion of a diiodomethane molecule induced by an X-ray free-electron laser pulse. *Phys. Chem. Chem. Phys.* **19,** 19707–19721 (2017).

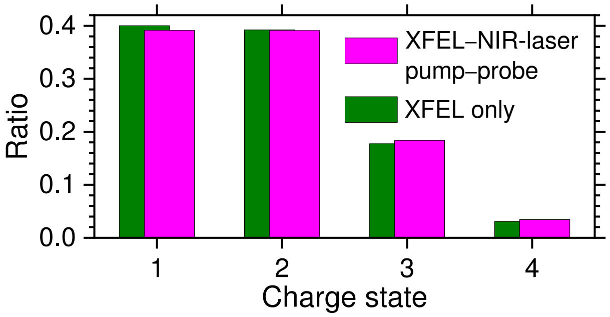

Supplementary Figure 1: **Charge state distributions of carbon ions.** Green bars show the ion yields relative to the total carbon ion yield, with XFEL irradiation only. Magenta bars show the ion yield ratios for the XFEL–NIR-laser pump–probe condition with a delay time window between -45 fs and $+125$ fs.

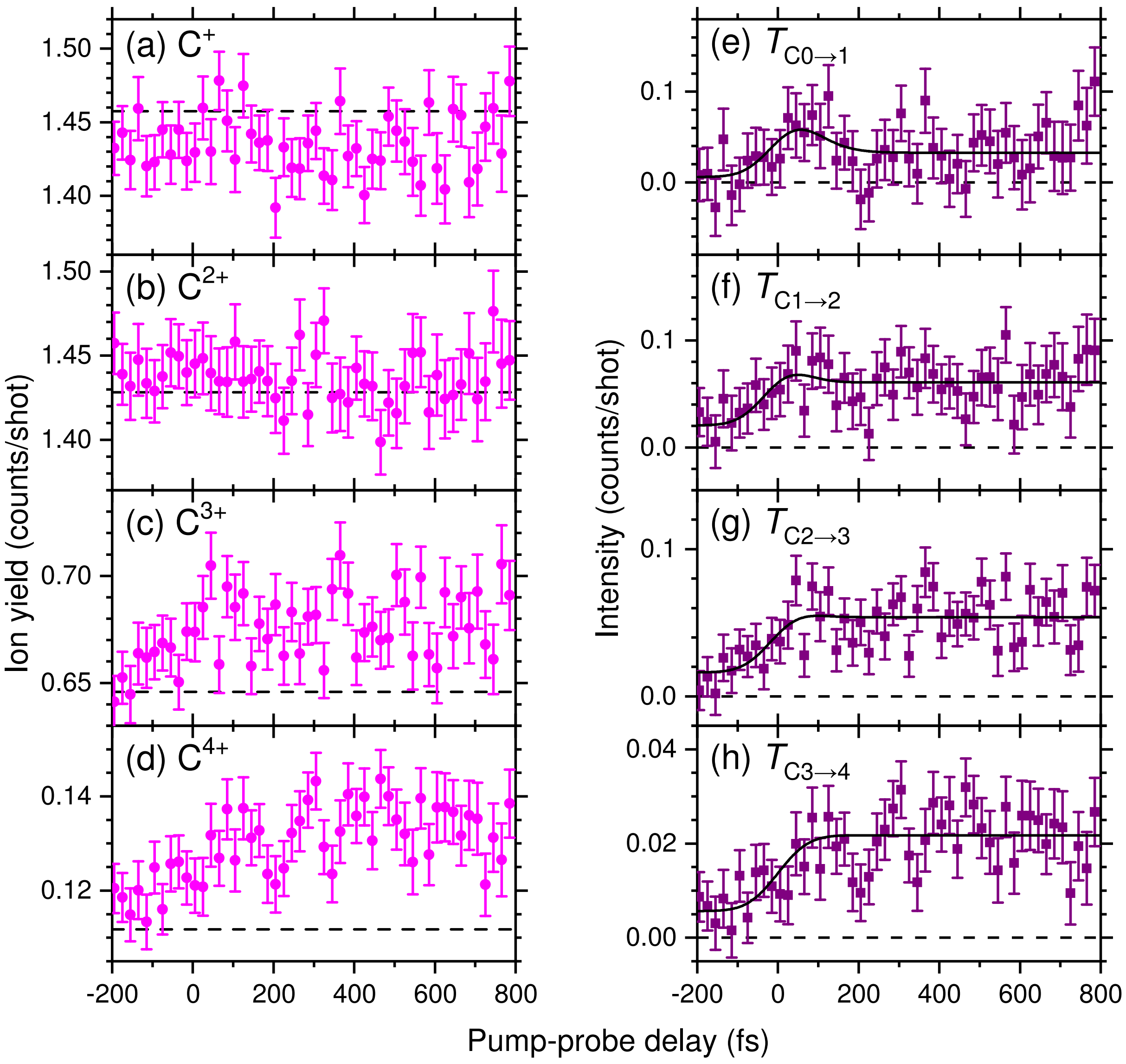

Supplementary Figure 2: **Time-evolution of the carbon ion yields.** (a)–(d) Carbon ion yields $Y_{\mathrm{C}q}(t)$ of C^{q+} as a function of the pump–probe delay. Magenta full circles indicate $Y_{\mathrm{C}q}(t)$, and horizontal dashed lines the baselines $B_{\mathrm{C}q}$ for the XFEL-only ionization. (e)–(h) $T_{\mathrm{C}q\to(q+1)}(t)$ for q = 0–3 obtained from Suppremetary Equation (1). Solid lines indicate the fitting results. Error bars are defined as standard deviation.

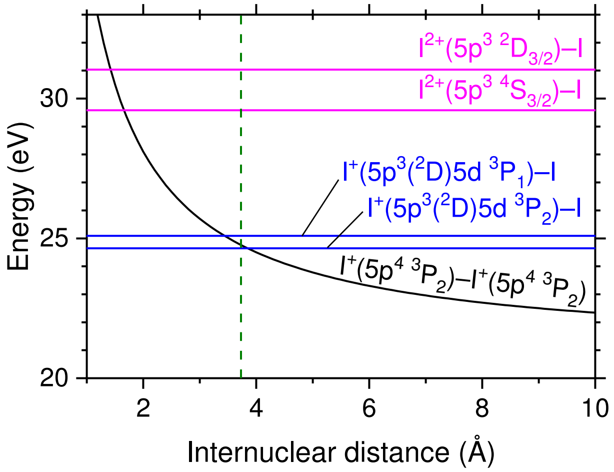

Supplementary Figure 3: **Potential energy curves for two iodine atoms to explain ICD.** Vertical broken line indicates a distance between two iodine atoms at equilibrium geometry of the CH_2I_2 molecule in the ground state. When $I^+(5p^3(^2D)5d\ ^3P_1)$ and neutral iodine atom are produced, ICD decay to $I^+(5p^4\ ^3P_2)$–$I^+(5p^4\ ^3P_2)$ is possible, even if at the equilibrium geometry of CH_2I_2. If internuclear distance slightly increases, ICD decay between $I^+(5p^3(^2D)5d\ ^3P_2)$ and neutral iodine is also possible. When the NIR-probe ionizes the excited I^+ before ICD occurs, the I^+ yield decreases while the I^{2+} yield increases. The I^{2+} state populated by the NIR-probe is $I^{2+}(5p^3\ ^2D)$.

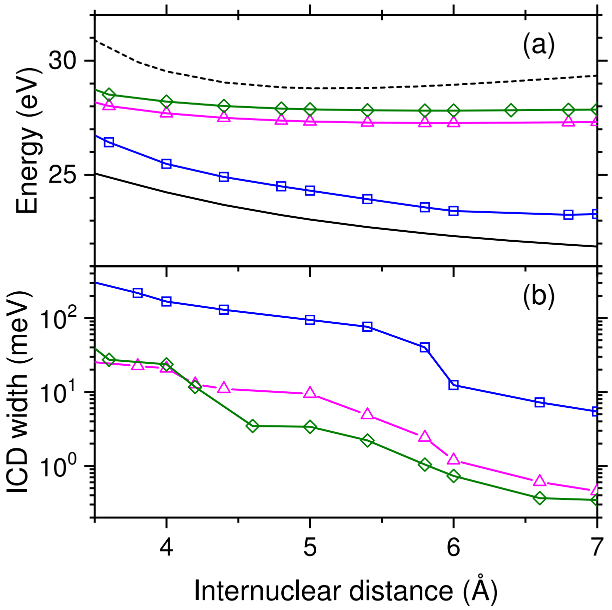

Supplementary Figure 4: **Calculated ionization energies and the ICD widths of some I–I^{+*}(5p^{-2}nl) ($^2\Pi$) ionization satellites.** (a): Ionization energies of three satellite states (squares, triangles, and diamonds) which lie between the lowest one-site double ionization I–I^{2+} threshold (broken line) and the lowest ICD final state (solid line). (b): ICD widths of the same three satellite states. A width of 10 meV corresponds to a lifetime of 66 fs.

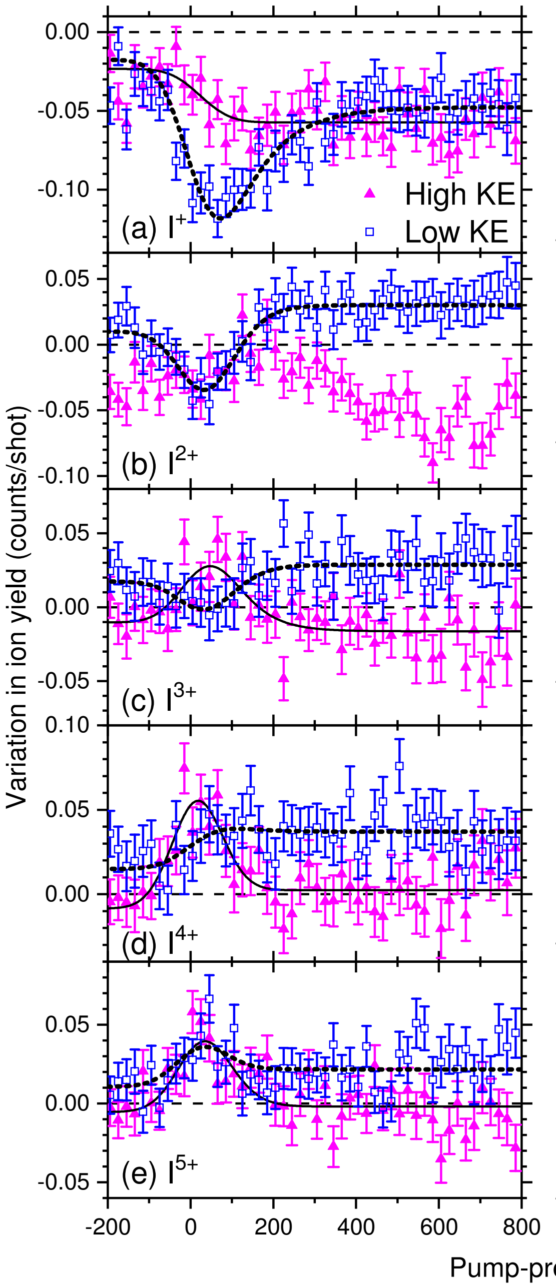

Supplementary Figure 5: **Time-evolution of the KE filtered ion yields.** The low KE (blue empty squares) and high KE (magenta full triangles) ion yields as a function of the pump–probe delay. The baseline for each component was subtracted from the data. Solid lines and dotted lines indicate the fitted curves for the high and low KE data, respectively. Error bars are defined as standard deviation.

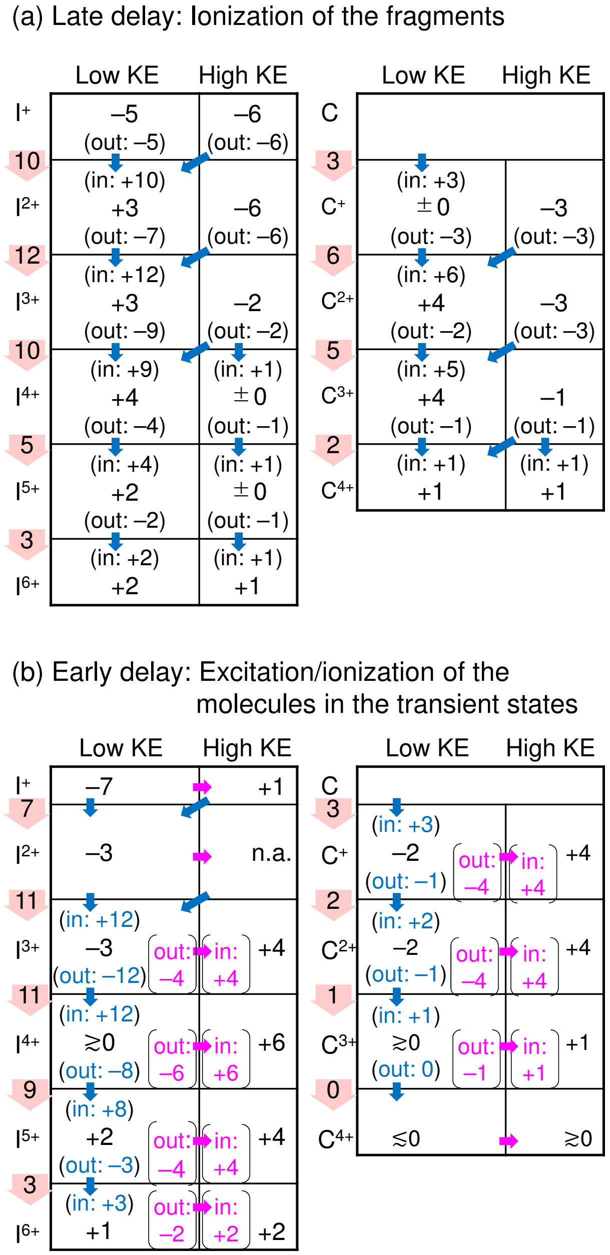

Supplementary Figure 6: **Flowchart.** (a) Flowchart for the change in ion yields due to the ionization of fragment ions by NIR laser absorption in the late delay region. (b) Flowchart for the change in ion yields and KE shifts due to the excitation/ionization of the molecular transient states by NIR laser absorption in the early delay region. The values are given in units of 0.01 counts/shot.
