# Supplementary material for: Real-time observation of X-ray-induced intramolecular and interatomic electronic decay in CH2I2
Source: Nat Commun. 2019 May 16;10:2186. doi: 10.1038/s41467-019-10060-z (PMC6522627; doi:10.1038/s41467-019-10060-z)
Supplement: Supplementary file 2 — Peer Review File [file 41467_2019_10060_MOESM2_ESM.pdf]

Editorial Note: This manuscript has been previously reviewed at another journal that is not operating a transparent peer review scheme. This document only contains reviewer comments and rebuttal letters for versions considered at *Nature Communications*. Mentions of prior referee reports have been redacted.

Reviewers' comments:

Reviewer #1 (Remarks to the Author):

The article by Fukuzawa et al. presents a time-resolved measurement of an Auger-ICD (Interatomic Coulombic Decay) cascade in the CH2I2 molecule in the gas-phase. The cascade is driven by an intense, ultrashort 5.5 keV XFEL pulse and probed by a time-delayed near-infrared (NIR) probe pulse, which increases the charge state of the fragment ions by +1. The experiment detects iodine and carbon ions using time-of-flight measurements and a position-sensitive detector.

The authors (and others) have in recent years demonstrated the importance of Auger cascades in the rapid XFEL-induced ionisation of samples such as rare-gas clusters and small molecules containing a heavy atom. This work has potential implications for single-particle x-ray imaging and the 'diffraction before destruction' dogma [Neutze et al., Nature 406 752 (2000)]. A complete and detailed understanding of the radiation damage processes from ultrafast and intense x-rays is desirable, and the presented experimental setup ingeniously combines a NIR probe pulse with the XFEL pump, causing +1 ionisation that gives a measurable time-resolved signal. It is a nice idea, but it should be noted that the obtained experimental data is rather coarse. It would have been helpful to have e.g. electron-ion coincidence data to support the analysis (especially with regard to the electron kinetic energies), but I accept the authors' assessment that this is practically impossible. Also, I was encouraged that the 100 fs time-scales observed are in reasonable agreement with previous ICD measurements. The analysis and interpretation rely heavily on included theoretical support, and a credible mechanism for the ICD is proposed using an energy diagram (as a function of internuclear distances) and calculated ionisation energies and ICD widths.

My remaining reservation about the manuscript relates to the first paragraph, which sets the work in the context of x-ray imaging. The authors point out that it is useful to understand the damage mechanisms that occur at XFELs and say that the damage may be severe in the presence of heavy atoms. However, many targets for x-ray imaging will be constructed from second and possibly third row elements, which are less strongly affected by the mechanisms observed by the authors - as acknowledged in their discussion of sulphur with another referee. The way that the paragraph is currently constructed, this aspect is potentially confusing: first, the example of complex molecular photocatalysts containing heavy atoms is mentioned, then the paragraph goes back to the general scenario by speaking about the value of non-invasive measurements. It then goes on to say that the effect of XFEL pulses is 'very severe' making it sound like this is a general statement rather than one relating to heavy elements specifically. Then finally it bounces back to the example of heavy atoms in the sentence 'In such photocatalysts deep inner-shell ionization of heavy atoms…'. The first paragraph would thus benefit from some editing to clarify that the observed damage mechanism predominantly occurs in heavy elements, and perhaps also some softening of the language (for instance 'is absolutely necessary' could be 'is necessary'; 'very severe' could be 'severe') which should aid clarity without diluting the message.

Reviewer #2 (Remarks to the Author):

In the resubmitted manuscript, the authors follow the advice given in the first round of review and make an effort to substantiate their claim of observation of the ICD step using theoretical input. Should this effort have been entirely successful, I would be happy to recommend the paper for publication in Nature Communications. At the moment, however, I still have got two issues with the ICD assignment:

1. Theory suggests a wide (order of magnitude) range of decay widths of different possible decaying states, while predicting (good news!) their relative stability with respect to I---I distance within the region of interest. Why should one disregard the state with the fast inter-atomic decay and assume that the two others, leading to slower decay, are the actually populated ones? What do we know about the possible population mechanisms of such states and can this be modelled, even if crudely? Assuming the fast decaying state was populated predominantly, would the present experiment be able to time-resolve such a decay?

2. How can we rule out the decay by energy transfer into the nuclear degrees of freedom? I tried to understand the reasoning of the authors given in response to Reviewer #4 and I could not. MIR kills ICD by ionising the excited electron, so the recombination energy needed for ICD is no longer there - this is fine. But why can't we imagine that MIR kills conical intersection/avoided crossing dynamics on the singly charged PESs by promoting the system to the doubly charged PESs?

Reviewer #3 (Remarks to the Author):

[Redacted] I am very pleased to see the fantastic improvements to this version. All of my (and similar ones of the previous reviewers) questions and concerns were aptly addressed. Thank you.

I recommend that this version of the manuscript and SI material, be published in Nature Communications.

Reviewer #4 (Remarks to the Author):

[Redacted] I am highly satisfied with these revisions and therefore recommend publication "as is" in Nature Communications.

Comments of the Reviewer #1

The article by Fukuzawa et al. presents a time-resolved measurement of an Auger-ICD (Interatomic Coulombic Decay) cascade in the CH2I2 molecule in the gas-phase. The cascade is driven by an intense, ultrashort 5.5 keV XFEL pulse and probed by a time-delayed near-infrared (NIR) probe pulse, which increases the charge state of the fragment ions by +1. The experiment detects iodine and carbon ions using time-of-flight measurements and a position-sensitive detector.

The authors (and others) have in recent years demonstrated the importance of Auger cascades in the rapid XFEL-induced ionisation of samples such as rare-gas clusters and small molecules containing a heavy atom. This work has potential implications for single-particle x-ray imaging and the 'diffraction before destruction' dogma [Neutze et al., Nature 406 752 (2000)]. A complete and detailed understanding of the radiation damage processes from ultrafast and intense x-rays is desirable, and the presented experimental setup ingeniously combines a NIR probe pulse with the XFEL pump, causing +1 ionisation that gives a measurable time-resolved signal. It is a nice idea, but it should be noted that the obtained experimental data is rather coarse. It would have been helpful to have e.g. electron-ion coincidence data to support the analysis (especially with regard to the electron kinetic energies), but I accept the authors' assessment that this is practically impossible. Also, I was encouraged that the 100 fs time-scales observed are in reasonable agreement with previous ICD measurements. The analysis and interpretation rely heavily on included theoretical support, and a credible mechanism for the ICD is proposed using an energy diagram (as a function of internuclear distances) and calculated ionisation energies and ICD widths.

My remaining reservation about the manuscript relates to the first paragraph, which sets the work in the context of x-ray imaging. The authors point out that it is useful to understand the damage mechanisms that occur at XFELs and say that the damage may be severe in the presence of heavy atoms. However, many targets for x-ray imaging will be constructed from second and possibly third row elements, which are less strongly affected by the mechanisms observed by the authors - as acknowledged in their discussion of sulphur with another referee. The way that the paragraph is currently constructed, this aspect is potentially confusing: first, the example of complex molecular photocatalysts containing heavy atoms is mentioned, then the paragraph goes back to the general scenario by speaking about the value of non-invasive measurements. It then goes on to say that the effect of XFEL pulses is 'very severe' making it sound like this is a general statement rather than one relating to heavy elements specifically. Then finally it bounces back to the example of heavy atoms in the sentence 'In such photocatalysts deep inner-shell ionization of heavy atoms…'. The first paragraph would thus benefit from some editing to clarify that the observed damage mechanism predominantly occurs in heavy elements, and perhaps also some softening of the language (for instance 'is absolutely necessary' could be 'is necessary'; 'very severe' could be 'severe') which should aid clarity without diluting the message.

Reply to the Reviewer #1:

Reviewer:

My remaining reservation about the manuscript relates to the first paragraph, which sets the work in the context of x-ray imaging. The authors point out that it is useful to understand the damage mechanisms that occur at XFELs and say that the damage may be severe in the presence of heavy atoms. However, many targets for x-ray imaging will be constructed from second and possibly third row elements, which are less strongly affected by the mechanisms observed by the authors - as acknowledged in their discussion of sulphur with another referee. The way that the paragraph is currently constructed, this aspect is potentially confusing: first, the example of complex molecular photocatalysts containing heavy atoms is mentioned, then the paragraph goes back to the general scenario by speaking about the value of non-invasive measurements. It then goes on to say that the effect of XFEL pulses is 'very severe' making it sound like this is a general statement rather than one relating to heavy elements specifically. Then finally it bounces back to the example of heavy atoms in the sentence 'In such photocatalysts deep inner-shell ionization of heavy atoms…'. The first paragraph would thus benefit from some editing to clarify that the observed damage mechanism predominantly occurs in heavy elements, and perhaps also some softening of the language (for instance 'is absolutely necessary' could be 'is necessary'; 'very severe' could be 'severe') which should aid clarity without diluting the message.

Reply:

Indeed, the introduction paragraph was a bit cumbersome. We have re-constructed it following the reviewer's suggestions and hope it is much clearer and easy to read now.

<u>Revised paragraph (1st paragraph of page 4)</u>

Understanding the details of the interaction between intense XFEL pulses[1,2] and matter is of paramount importance for their numerous applications, including single-particle structural determination by coherent X-ray imaging[3–5] and structural dynamics tracking in molecules by time-resolved X-ray spectroscopy and diffraction[6–12]. Another powerful method made available by the new XFELs is serial femtosecond crystallography (SFX)[13–15]. It allows structural determination of proteins, especially membrane proteins, which are difficult to crystalize. However, an in-depth knowledge of the radiation damage caused by the XFEL irradiation[16–19] is necessary for the implementation of the above mentioned XFEL imaging methods. In imaging applications, the primary purpose of the XFEL pulses is to probe the structure of the object, ideally as noninvasively as possible. The impact of the extremely concentrated energy of the XFEL pulses on the target materials is, however, severe and still not fully characterized, in particular, the changes induced in the electronic cloud of the sample over the course of the imaging process. A prominent example is the application of XFEL imaging to complex molecular photocatalysts containing heavy metal atoms. In such photocatalysts, the deep inner-shell ionization of heavy atoms releases many electrons via cascading electronic relaxation processes and, due to the Coulomb repulsion between highly charged atomic sites, eventually results in the fast destruction of chemical bonds and consequently in rapidly developing radiation damage at the molecular level. Detailed knowledge of the underlying mechanisms can be obtained through real-time observations of the structural changes and of the charge states created by the XFEL pulses interrogating the sample. To better understand the molecular-level radiation damage in polynuclear

complexes containing heavy atoms, we studied the CH_2I_2 molecule, obtained by the substitution of two hydrogens with two iodine atoms in methane, which may be regarded as the simplest model system of that type.

Comments of the Reviewer #2

In the resubmitted manuscript, the authors follow the advice given in the first round of review and make an effort to substantiate their claim of observation of the ICD step using theoretical input. Should this effort have been entirely successful, I would be happy to recommend the paper for publication in Nature Communications. At the moment, however, I still have got two issues with the ICD assignment:

1. Theory suggests a wide (order of magnitude) range of decay widths of different possible decaying states, while predicting (good news!) their relative stability with respect to I---I distance within the region of interest. Why should one disregard the state with the fast inter-atomic decay and assume that the two others, leading to slower decay, are the actually populated ones? What do we know about the possible population mechanisms of such states and can this be modelled, even if crudely? Assuming the fast decaying state was populated predominantly, would the present experiment be able to time-resolve such a decay?

2. How can we rule out the decay by energy transfer into the nuclear degrees of freedom? I tried to understand the reasoning of the authors given in response to Reviewer #4 and I could not. MIR kills ICD by ionising the excited electron, so the recombination energy needed for ICD is no longer there - this is fine. But why can't we imagine that MIR kills conical intersection/avoided crossing dynamics on the singly charged PESs by promoting the system to the doubly charged PESs?

Reply to the Reviewer #2:

Reviewer:

1. Theory suggests a wide (order of magnitude) range of decay widths of different possible decaying states, while predicting (good news!) their relative stability with respect to I---I distance within the region of interest. Why should one disregard the state with the fast inter-atomic decay and assume that the two others, leading to slower decay, are the actually populated ones? What do we know about the possible population mechanisms of such states and can this be modelled, even if crudely?

Reply:

In principle, there is a number of possible mechanisms that might lead to the population of initial ICD states. Those can be, for example, various radiative-Auger cascades involving valence electrons from the C-I bond producing $CH_2^{n+}I_2^{+*}$, leading to a fast disintegration of the molecule into CH_2^{n+} and $I^{+*}\cdots I$. It is very difficult, if not impossible, to quantitatively model the mechanisms populating the ICD initial states and thus determine the branching ratios of the different ICD channels. Moreover, since all of them lead to the same observable, namely two I^+ ions, they will be hardly discernable in the experiment. If the fast ICD channels are not overwhelmingly dominant, they will be masked by the slower transitions in the time-dependent ion yield (see, PRL **118**, 033202 (2017)). Since, most likely, many ICD transitions contribute, and also the distribution of inter-iodine distances is expected to be rather wide, the time-dependent ion yield function will appear as a single relaxation with an effective (averaged) time constant, as observed in our measurements.

Reviewer:

Assuming the fast decaying state was populated predominantly, would the present experiment be able to time-resolve such a decay?

Reply:

In principle, yes. Our time-resolution is good enough to observe decay times of ~20 fs, as we clearly demonstrated for the higher charge states. However, as pointed out above, in order to be able to discern the multi-exponential character of the ion yield function, the fast transition should be by far the dominant relaxation channel.

Reviewer:

2. How can we rule out the decay by energy transfer into the nuclear degrees of freedom? I tried to understand the reasoning of the authors given in response to Reviewer #4 and I could not. MIR kills ICD by ionising the excited electron, so the recombination energy needed for ICD is no longer there - this is fine. But why can't we imagine that MIR kills conical intersection/avoided crossing dynamics on the singly charged PESs by promoting the system to the doubly charged PESs?

Reply:

We do not claim that energy transfer to the nuclear degrees of freedom does not occur. However, this cannot explain our experimental observations. Let us assume that NIR kills conical intersection dynamics on the singly charged PESs by promoting the system to the doubly charged PESs, as the reviewer considered. If the NIR pulses do not quench the process, I^+ and other neutral fragments might be produced (if the system disintegrates at all). When the NIR promotes the system to a doubly charged species, the molecule will most probably break-up into I^+ and other ionic and neutral fragments, rather than into I^{2+} and neutrals. Thus, this process cannot explain our experimental finding: NIR reduces I^+ yield. Reduction of the I^+ yield might indeed be observed if the NIR could quench avoided crossing dynamics in the $I^{+*}\cdots I$, after initial disintegration of the molecule. However, even the lowest $I^{+*}\cdots I$ state lies far above the ground $I^+\cdots I$ state, and thus no such avoided crossing dynamics could be expected.

Comments of the Reviewer #3

[Redacted] I am very pleased to see the fantastic improvements to this version. All of my (and similar ones of the previous reviewers) questions and concerns were aptly addressed. Thank you.

I recommend that this version of the manuscript and SI material, be published in Nature Communications.

Reply to the Reviewer #3:

We would like to thank the reviewer for the careful reading and report.

Comments of the Reviewer #4

[Redacted] I am highly satisfied with these revisions and therefore recommend publication "as is" in Nature Communications.

Reply to the Reviewer #3:

We would like to thank the reviewer for the careful reading and report.

REVIEWERS' COMMENTS:

Reviewer #1 (Remarks to the Author):

The authors have taken all previous comments on board, and replied to all questions. I recommend publication in Nature Communications.

Reviewer #2 (Remarks to the Author):

In their response, the authors have addressed the main concern I had about the ICD assignment. I am very glad to recommend the manuscript for publication in Nature Communications in its present form.

Comments of the Reviewer #1

The authors have taken all previous comments on board, and replied to all questions. I recommend publication in Nature Communications.

Reply to the Reviewer #1:

We would like to thank the reviewer for the recommendation of the publication in Nature Communications.

Comments of the Reviewer #2

In their response, the authors have addressed the main concern I had about the ICD assignment. I am very glad to recommend the manuscript for publication in Nature Communications in its present form.

Reply to the Reviewer #2:

We would like to thank the reviewer for the recommendation of the publication in Nature Communications.
